# Pain Facilitation Brain Regions Activated by Nalbuphine Are Revealed by Pharmacological fMRI

**DOI:** 10.1371/journal.pone.0050169

**Published:** 2013-01-08

**Authors:** Robert Gear, Lino Becerra, Jaymin Upadhyay, James Bishop, Diana Wallin, Gautam Pendse, Jon Levine, David Borsook

**Affiliations:** 1 Department of Oral & Maxillofacial Surgery, University of California San Francisco, San Francisco, California, United States of America; 2 Center for Pain and the Brain, Harvard Medical School, Boston, Massachusetts, United States of America; 3 Brain Imaging Center, McLean Hospital, Belmont, Massachusetts, United States of America; 4 Harvard Medical School, Boston, Massachusetts, United States of America; 5 Departments of Radiology and Psychiatry, Massachusetts General Hospital, Harvard Medical School, Boston, Massachusetts, United States of America; 6 Department of Medicine, University of California San Francisco, San Francisco, California, United States of America; University of Ulster, United Kingdom

## Abstract

Nalbuphine, an agonist-antagonist kappa-opioid, produces brief analgesia followed by enhanced pain/hyperalgesia in male postsurgical patients. However, it produces profound analgesia without pain enhancement when co-administration with low dose naloxone. To examine the effect of nalbuphine or nalbuphine plus naloxone on activity in brain regions that may explain these differences, we employed pharmacological magnetic resonance imaging (phMRI) in a double blind cross-over study with 13 healthy male volunteers. In separate imaging sessions subjects were administered nalbuphine (5 mg/70 kg) preceded by either saline (Sal-Nalb) or naloxone 0.4 mg (Nalox-Nalb). Blood oxygen level-dependent (BOLD) activation maps followed by contrast and connectivity analyses revealed marked differences. Sal-Nalb produced significantly increased activity in 60 brain regions and decreased activity in 9; in contrast, Nalox-Nalb activated only 14 regions and deactivated only 3. Nalbuphine, like morphine in a previous study, attenuated activity in the inferior orbital cortex, and, like noxious stimulation, increased activity in temporal cortex, insula, pulvinar, caudate, and pons. Co-administration/pretreatment of naloxone selectively blocked activity in pulvinar, pons and posterior insula. Nalbuphine induced functional connectivity between caudate and regions in the frontal, occipital, temporal, insular, middle cingulate cortices, and putamen; naloxone co-admistration reduced all connectivity to non-significant levels, and, like phMRI measures of morphine, increased activation in other areas (e.g., putamen). Naloxone pretreatment to nalbuphine produced changes in brain activity possess characteristics of both analgesia and algesia; naloxone selectively blocks activity in areas associated with algesia. Given these findings, we suggest that nalbuphine interacts with a pain salience system, which can modulate perceived pain intensity.

## Introduction

In clinical studies of the analgesic efficacy of agonist-antagonist kappa opioids we found that all three clinically available agents in this class (viz., nalbuphine, pentazocine, and butorphanol) produce sexually dimorphic analgesia [1], [2], with males experiencing significantly less analgesia than females. Although the reasons for this sex difference have not been determined, a placebo-controlled dose response study of the analgesic effect of nalbuphine in patients experiencing moderate to severe post-operative pain showed that males, and not females, receiving the lowest dose of nalbuphine (5 mg), reported significantly *greater* pain than those receiving placebo [3], suggesting the presence of a pain-facilitating mechanism in males, not present or significantly diminished in females. Subsequently, we found that co-administration of the opioid antagonist naloxone, at low dose, abolished pain facilitation in males, resulting in profound analgesia that was very similar to that produced by nalbuphine in females [4]. Based on these findings we hypothesized that nalbuphine has pain-facilitating as well as analgesic effects produced by action at different receptors in the brain, an “analgesia” receptor, likely the κ-opioid receptor [5] and a “pain-facilitating” receptor, and that the latter, which is predominantly found in males, is more sensitive to naloxone. This model could explain the ability of naloxone to enhance analgesia by selectively blocking the pain-facilitating effect of nalbuphine.

The present study investigated putative analgesia and pain-facilitation brain circuitry affected by nalbuphine, in males. Since a radio-ligand for κ-opioid receptors is not currently available for use in human positron emission tomography (PET) or single positron emission tomography (SPECT) studies, we employed pharmacological magnetic resonance imaging (phMRI), a form of functional magnetic resonance imaging (fMRI), to identify regions of the brain whose blood oxygen level-dependent (BOLD) signals are increased or decreased by nalbuphine in subjects pretreated with either saline or naloxone. Important to the present study, phMRI (a measure of BOLD activation/brain function as a direct consequence of drug induced activity) is a technique that enables the measures of activity in brain regions affected by analgesics and other drugs without sensory stimulation [6], [7]. We have previously used phMRI to study other opioid agonists and antagonists including morphine [8] and naloxone [9], and most recently buprenorphine [10], demonstrating the utility of this approach to evaluate drug action on brain circuits.

In the present study male volunteers were administered nalbuphine preceded by vehicle saline or nalbuphine preceded by naloxone, in a double blind, crossover, two-session design to test the hypothesis that nalbuphine affects activity in brain regions and circuits related to pain-facilitation, as well as analgesia, and its effects on sites and circuits involved in possible pain facilitation are attenuated by naloxone pretreatment/co-administration.

## Methods

To investigate the differences in phMRI brain activation produced by nalbuphine and nalbuphine in combination with naloxone, each subject was scanned twice in a cross-over double blind study. Thus, each subject received nalbuphine infusions on each trial, once with saline pretreatment (Sal-Nalb) and once with naloxone pretreatment (Nalox-Nalb).

### Subjects

#### Recruitment

The McLean Hospital Institutional Review Board approved the study. The study also complied with the guidelines of the Helsinki Accord and International Association for the Study of Pain (IASP) for experimental pain research in humans. Fifteen healthy male volunteers (age 24.07±2.56 (mean ± SD)) were recruited through advertisements in local newspapers, flyers on local college campuses, and on recruiting websites (Partners research site (CRNet), and Craig's List (http://boston.craigslist.org/). All subjects signed a written informed consent to participate in the study.

#### Enrollment

Healthy right-handed male candidates 18–45 years of age attended a screening session where compliance with inclusion/exclusion criteria was evaluated and a full medical examination including was performed by a board certified neurologist. Inclusionary criteria. Candidates meeting any of the following criteria were excluded from participation in the study: current use of prescribed medications, BDI score >11, history of opioid abuse, history of smoking within the past year, claustrophobia, significant medical problems, significant alcohol intake (five or more glasses/week), history of allergy or untoward reaction to anticonvulsants, metal implants of any type, weight greater than 130 kg. An FDA-approved urine toxicology screen (7 Drug InstaStrip Drug Screen Test, Cortez Diagnostics, Calabasas, CA), which tested for barbiturates, benzodiazepines, amphetamine, cocaine, tetrahydrocannabinol, phencyclidine and opioids, was performed prior to each MR session. None of the subjects had a positive drug screen. Thirteen of the 15 subjects (three Asian, one black, 9 white, aged 24±2.56 years (mean ± s.d), weight 79±16.4 kg, completed both scanning sessions.

#### Subject Preparation

All subjects were instructed to refrain from eating starting eight hours before the scan but were allowed to consume clear liquids. Subjects were scheduled for their second scan two weeks after the first.

### Study Design

#### Drug Administration

**Figure 1:**
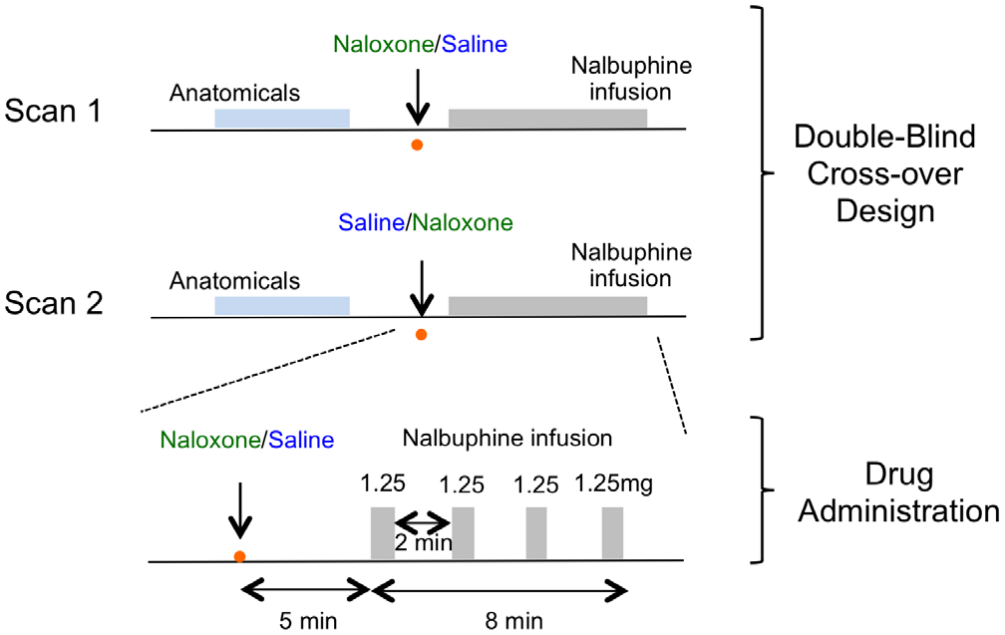
Drug administration protocol. After the initial anatomical scans were obtained, either naloxone or saline was infused for Scan 1; for Scan 2 the other drug was infused. Nalbuphine infusion was then started five minutes later. One quarter of the nalbuphine dose was administered every other minute until the full amount was delivered by eight minutes.

Naloxone and nalbuphine (both from Hospira Inc., Lake Forest, IL) were administered through contralateral intravenous lines placed in the antecubital region of each arm. Naloxone (0.4 mg) or saline was followed 5, 7, 9 and 11 minutes later by a divided dose of nalbuphine (5 mg/70 kg) administered through the access port in the left arm in 2 ml boluses at 0.1 ml/s (Fig. 1). Doses were chosen on the basis of our previous clinical studies [3], [4], [11]. Nalbuphine administration was carried out with an MRI-compatible microinjector (Medrad Spectris, Colombus, OH).

#### Randomization

To neutralize any effect of treatment order, about half of the subjects received saline first and the other half received naloxone first, in a randomized, double-blinded, crossover design. Subjects as well as team members involved in drug administration and initial analysis were blinded to drug group assignment.

### Scanning

Anatomical scans were acquired prior to drug administration (Fig. 1). Infusion scanning began immediately after infusion of naloxone or saline (five minutes before infusion of the first bolus of nalbuphine).

#### Data Acquisition

Data were collected on a 3T Siemens Trio scanner with an 8-channel phased array head coil (Erlangen, Germany). phMRI data were collected using a gradient echo-echo planar pulse sequence (GE-EPI) at 3.5×3.5×3.5 mm^3^ resolution.

#### GE-EPI Parameters

Time of Repetition (TR) = 2500 msec, Time of Echo (TE) = 30 msec, Field of View (FOV) = 224×224, Flip Angle (FA) = 90°, number of Slices = 41 axial slices and number of Volumes = 600. The acquisition time for the infusion scan was 25 min 5 sec. T_1_-weighted structural images were acquired using a 3-D magnetization-prepared rapid gradient echo (MPRAGE) sequence at a resolution of 1.33×1.0×1.0 mm^3^. MPRAGE Parameters: TR = 2100 msec, TE = 2.74 msec, Time of Inversion (TI) = 1100 msec, FA = 12°, number of Slices = 128 sagittal slices.

### Ancillary Measures

#### Hedonic Ratings

Hedonic ratings were recorded following nalbuphine administration. Prior to the scan subjects were instructed on how to rate their hedonic experiences using a dial controlled by the right hand and a visual analog scale (VAS) display that could be seen from within the bore of the magnet. The VAS anchors were ‘neutral’ in the middle of the scale, ‘max euphoria’ on the right, and ‘max dysphoria’ on the left.

#### Physiological Measures

Throughout the period of the scans, pulse oximetry was employed to monitor heart rate (HR) and blood oxygen saturation (SpO_2_), and a nasal cannula was placed to monitor respiratory rate (RR) and end tidal CO_2_.

### Imaging Data Analysis

#### Preprocessing

*Anatomical data:* High resolution anatomical images for each subject were bias-corrected and segmented into white matter (WM), gray matter (GM) and cerebrospinal fluid (CSF) using FAST [12].

*phMRI data:* Preprocessing was performed using tools from the FMRIB Software Library (FSL) (http://www.fmrib.ox.ac.uk/fsl/). The first 2 volumes were removed from each subject's phMRI dataset to allow the MR signal to stabilize.

Preprocessing steps on the remaining 4D volume included: (1) motion-correction using MCFLIRT [13]; (2) brain extraction using BET [14]; (3) spatial smoothing using an isotropic Gaussian kernel of 5 mm FWHM; (4) grand mean intensity normalization of the entire 4D dataset by a single multiplicative factor; (5) registration of the functional space template to the anatomical space and the MNI 152 space using an affine transform with 12 degrees of freedom via FLIRT [13]; and (6) creation of the WM and CSF masks in functional space for each subject using the functional space affine transformation.

The phMRI data was not high pass filtered since our signal of interest is a low frequency infusion response. Preprocessed phMRI was inspected for scanner artifacts using a linear independent component analysis (ICA) based approach [15]. Confounding artifacts were removed from the phMRI before statistical analysis.

### Infusion analysis

#### Single subject

Analysis was performed using a generalized linear model (GLM) with Gaussian noise. The ideal infusion response (infusion EV) was modeled as a ramped unit step function with a 5 min baseline, a ramp going from 5 min to 13 min and a plateau thereafter. The infusion EV was not convoluted to a hemodynamic response function. The key statistic of interest is the coefficient/amplitude of the “infusion EV” in the GLM. Since the onset of the ramp can be variable depending on local pharmacodynamics, we included additional singular value decomposition regressors to enable unbiased estimation [16] of the infusion amplitude. Further, linear drift and subject specific average time courses extracted from the WM and CSF were included as covariates of no interest in the GLM. The statistical outputs from the single subject analysis were transformed to the MNI 152 space using precomputed affine transformations.

#### Group level

For all group-level phMRI analyses, single subject GLM statistics for the amplitude of the “infusion EV” were entered into a paired mixed-effects group-level GLM analysis using a paired *t*-test design with FLAME [17]. The contrasts of interest were Sal-Nalb>Nalox-Nalb and Sal-Nalb<Nalox-Nalb.

### Functional connectivity analysis

Differences in functional connectivity (Fc) between the Nalox-Nalb and Sal-Nalb conditions for specific brain structures were calculated. Only phMRI data from the last 5 min were analyzed for Fc because the drug effects were in a steady state during this time. A seed-based analysis was performed as described below:

#### Region of interest selection

Biologically relevant regions of interest (ROIs) were defined in the standard space MNI 152 template and subsequently linearly transformed into the subject-specific fMRI space for functional connectivity analysis.

ROIs that showed significant differences in Fc were selected on the basis of evidence for such changes in observed phMRI infusion responses between Sal-Nalb and Nalox-Nalb treatments.

#### Single subject Fc analysis

Functional connectivity analysis was performed utilizing a partial correlation coefficient approach between all brain voxels and the seed time course given the average WM, average CSF, and the linear drift as nuisance covariates. The seed time course was defined as the averaged time course of all the voxels within an ROI. We used partial correlation because it was recently shown to have good sensitivity in detecting network connections [18].

#### Group-level Fc analysis

The raw partial correlation coefficient maps from single subject analysis were variance-stabilized using a Fisher z-transform and transformed to the MNI 152 space using precomputed affine transformations. The resulting z-transformed maps were entered into a random effects group-level GLM analysis using a paired *t*-test design. As in the case of infusion analysis, the contrasts of interest were Sal-Nalb>Nalox-Nalb and Nalox-Nalb->Sal-Nalb.

#### Statistical inference and thresholding

The z-statistic maps for each contrast in the infusion analyses as well as the functional connectivity analyses were subjected to alternative hypothesis testing using Gaussian mixture modeling [19]. In each case, the mixture model was spatially regularized using a Markov random field (MRF) that was a soft-max prior on the class labels [20]. This prior encourages spatially neighboring voxels to have similar labels. The mixture model parameters as well as the MRF parameter were adaptively estimated from the data using iterated conditional modes (ICM) [20]. The posterior probability maps (PPMs) giving the “activation” probability of a voxel conditional on the estimated labels in its neighborhood and the observed data were created and thresholded at PPM>0.5 to detect “activation”.

### PhMRI comparisons with morphine and naloxone

In order to infer biological relevance from the differences in the patterns of brain activation by Sal-Nalb and Nalox-Nalb, we compared the results of the current study with those of previous studies of morphine [8] and naloxone [9], which employed the same infusion protocol as that for the actual Nalbuphine administration.

## Results

### Psychophysical ratings and physiological measures

There were no significant differences between the two groups in euphoria-dysphoria scores, respiratory rate, end tidal CO_2_, or heart rate (data not shown).

### phMRI infusion responses

#### PhMRI BOLD responses

**Figure 2:**
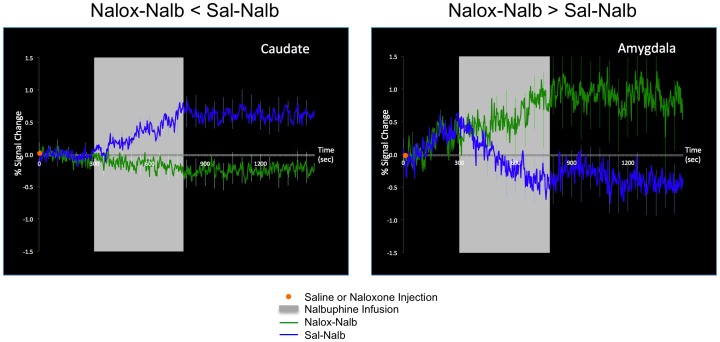
Examples of infusion-initiated BOLD signal changes. Shown is an example in which the Nalb-Sal treatment induced greater activation (i.e., caudate), and an example in which the Nalb-Nalox treatment induced greater activation (i.e., amygdala).

**Figure 3:**
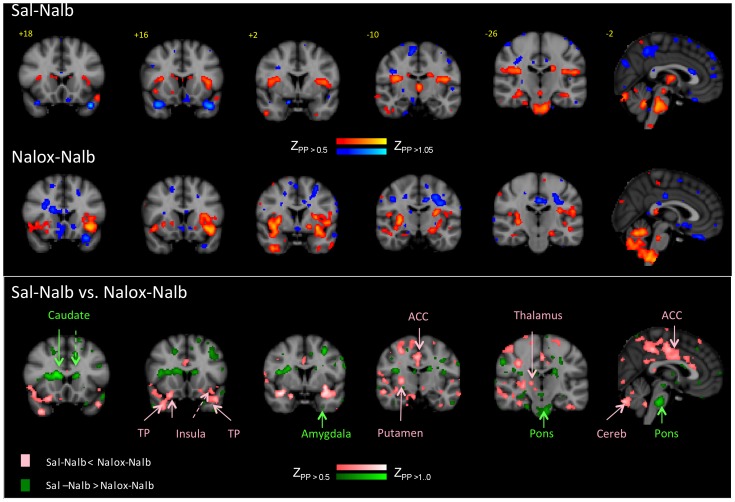
BOLD phMRI activation maps. *Top*: BOLD activation maps for Nalb-Sal (see Table 1); *middle: BOLD activation maps for* Nalb-Nalox infusions (see Table 2). *Bottom:* Contrast maps for Nalb-Sal>Nalb-Nalox and Nalb-Nalox>Nalb-Sal (see Table 3).

Sal-Nalb and Nalox-Nalb produced significant changes in activity in many brain regions. Representative examples of the time courses of changes in activation are shown in **Fig. 2**. In caudate and anterior insula Sal-Nalb induced greater signal changes than did Nalb-Nalox; in contrast, the effects were opposite in the amygdala and the middle cingulate cortex (MCC). Except for the MCC, these changes commenced soon after nalbuphine infusion started. These signal changes exemplify the data used to derive the statistically significant signal changes for regional activation shown in **Tables 1** **,** **2** **, and** **3** and mapped in **Fig. 3**.

**Table 1:** Nalb-Sal-induced changes in BOLD activity.

			Coordinates (mm)			Volume
Brain Region	Lat.	Z-stat	x	y	Z	cm^3^
***Increased Response***						
Cortical						
Occipital						
Rolandic Operculum	R	3.48	44	0	14	1.064
Rolandic Operculum	L	3.07	−44	−12	18	1.168
Rolandic Operculum	R	3.78	54	−26	20	4.536
Calcarine	R	3.42	30	−52	10	0.832
Calcarine	R	3.72	30	−58	10	1.048
Middle	R	4.07	46	−76	24	2.66
Middle	R	4.06	42	−82	24	2.552
Temporal						
Pole Superior	L	2.86	−50	18	−16	0.432
Inferior	R	2.84	50	2	−36	0.976
Inferior	L	4.23	−52	−6	−34	1.16
Superior	R	3.84	54	−38	14	1.08
Superior	R	3.30	50	−40	12	1.216
Inferior	L	3.18	−46	−48	−18	0.784
Inferior	L	3.35	−50	−50	−16	0.288
Fusiform	L	4.12	−46	−54	−18	0.996
Insula						
Insula Anterior	L	3.38	−38	8	8	1.808
Insula Anterior	R	2.97	38	4	12	2.272
Insula Anterior	L	3.75	−42	2	10	1.504
Insula Anterior	L	3.07	−36	2	12	0.224
Insula Posterior	L	2.80	−32	−2	12	0.576
Insula Posterior	R	3.55	34	−4	16	0.672
Insula Posterior	L	2.89	−36	−12	18	0.808
Sub-Cortical						
Thalamus	L	3.15	−2	−14	2	1.008
Thalamus (pulvinar)	R	4.56	10	−30	8	0.688
Thalamus (pulvinar)	L	3.86	−10	−30	10	0.656
Caudate	L	2.99	−18	−18	24	0.512
Hippocampus	R	3.66	32	−30	−10	1.672
Hippocampus	L	3.45	−32	−32	−12	2.152
Hippocampus	R	4.04	30	−34	−8	0.728
Hippocampus	L	3.81	−14	−36	2	0.864
Hippocampus	L	2.88	−34	−36	−8	0.576
Hippocampus	L	3.61	−22	−38	0	1.136
Hippocampus	L	3.72	−28	−38	−4	1.232
Brainstem/Cerebellum						
Pons	L	3.20	−6	−18	−34	0.504
Pons	B	2.61	0	−22	−40	0.6
Pons	L	2.57	−6	−22	−38	0.312
Pons	B	2.80	0	−24	−32	0.376
msn	L	4.31	−20	−32	−36	5.128
Cerebellum 8	L	3.48	−20	−38	−48	0.4
Crus 4 5	L	2.93	−16	−38	−30	0.288
Cerebellum 10	L	3.40	−20	−40	−44	0.552
Cerebellum 9	R	3.48	36	−42	−48	0.6
Cerebellum 4 5	R	2.84	16	−42	−28	0.632
Cerebellum Crus1	R	3.51	20	−46	−40	1.28
Cerebellum 9	R	4.07	40	−48	−42	0.928
Cerebellum 9	L	3.58	−2	−48	−44	0.352
Cerebellum 7b	R	3.58	4	−58	−46	1.624
Vermis 9	R	2.80	24	−52	−30	0.496
Vermis 9	L	2.83	−22	−52	−32	2.112
Cerebellum 8	L	3.88	−30	−52	−42	1.664
Cerebellum 8	R	2.86	36	−52	−46	0.568
Cerebellum 8	L	3.86	−30	−52	−54	0.888
Cerebellum Crus1	R	3.27	36	−54	−34	0.536
Cerebellum 8	L	3.84	−16	−56	−48	0.56
Cerebellum 4 5	L	3.05	−6	−56	−12	0.536
Cerebellum 8	L	3.19	−14	−60	−58	0.872
Cerebellum 8	L	3.93	−24	−62	−50	2.328
Cerebellum Crus1	R	3.46	24	−64	−34	1.616
Cerebellum Crus1	L	4.13	−44	−74	−30	2.688
Cerebellum I–IV	L	2.91	−14	−44	−26	0.48
***Decreased Response***						
Cortical						
Frontal						
Middle	R	−2.46	14	62	−2	4.296
Inferior Orbital	R	−3.48	34	16	−24	0.68
Parietal						
Postcentral (S1)	R	−3.63	14	−40	78	0.936
Postcentral (S1)	R	−3.34	20	−40	76	1.312
Precuneus	B	−2.86	0	−48	44	1.464
Temporal						
Pole Superior	R	−3.55	38	12	−24	0.664
Pole Superior	L	−3.44	−38	10	−26	2.168
Cerebellum						
Cerebellum 3	L	−3.12	−14	−32	−24	1.032
Cerebellum 4 5	R	−2.87	10	−40	−12	2.24

Group averages for significant Nalb-Sal-induced changes in BOLD activity. General brain regions, given in the left column, may contain several significantly activated/deactivated regions, each listed with its MNI-152 coordinates. See Fig. 3 (“Nalb-Sal”) for maps depicting the data in this table. “Lat.” indicates the brain laterality (i.e., R = right side, L = left side). Z-stat is given for each region. Coordinates: x = mm right (positive values) or left (negative values); y = mm anterior (positive values) or posterior (negative values); and z = mm superior (positive values) or inferior (negative values). Volume is the volume of the region showing significant BOLD activation or deactivation.

**Table 2:** Nalb-Nalox-induced changes in BOLD activity.

			Coordinates (mm)			Volume
Brain Region	Lat.	Z-stat	x	Y	z	cm^3^
***Increased Response***						
Cortical						
Temporal						
Lingual	L	3.41	−26	−48	−8	5.048
Insula						
Insula Anterior	L	3.78	−36	18	−8	1.36
Insula Anterior	L	4.42	−38	10	−12	3.216
Insula Anterior	R	3.22	42	8	−6	2.072
Insula Anterior	R	3.72	36	4	−6	6.904
Insula Anterior	R	3.76	38	0	−6	0.96
Sub-Cortical						
Putamen	R	3.34	30	−8	2	3.448
Brainstem/Cerebellum						
spV	L	4.42	−2	−44	−58	9.96
Cerebellum 4 5	R	3.81	14	−50	−20	1.64
Vermis 4 5	R	4.65	6	−54	−18	2.48
Vermis 6	B	3.51	0	−62	−20	1.208
Cerebellum 8	L	4.39	−18	−60	−52	4.632
Cerebellum 9	L	3.82	−18	−44	−60	10.288
Cerebellum 10	L	3.14	−14	−40	−44	1.064
***Decreased Response***						
Cortical						
Frontal						
Superior	R	−3.18	14	52	0	0.592
Inferior Orbital	L	−2.80	−28	24	−22	1.832
Occipital						
Superior	L	−2.32	−8	−96	6	0.624

Group averages for significant Nalb-Nalox-induced changes in BOLD activity. See caption for Table 1 for explanation of the columns. See Fig. 3 (“Nalb-Nalox”) for maps depicting the data in this table. Note that no subcortical, brainstem, or cerebellar regions showed significantly decreased activity.

**Table 3:** Contrast analyses for Nalb-Sal vs Nalb-Nalox.

			Coordinates (mm)			Volume
Brain Region	Lat.	Z-stat	x	Y	z	cm^3^
***Nalb-Sal<Nalb-Nalox***						
Cortical						
Frontal						
Superior Orbital	R	3.83	16	64	0	0.808
Superior	R	3.82	12	62	0	0.6
Superior	R	3.40	12	60	4	1.928
Inferior Orbital	R	3.30	44	32	−8	6.568
Parietal						
Postcentral	R	3.46	24	−42	72	19.36
Occipital						
Cuneus	R	3.50	18	−76	40	1.72
Temporal						
Pole Middle	R	3.54	36	16	−38	1.512
Pole Superior	L	4.13	−42	12	−26	1.424
Pole Superior	L	3.19	−34	8	−26	0.664
Superior Temporal Lobe	L	3.78	−42	−2	−20	1.224
Cingulum						
Middle	R	3.03	2	−12	42	4.456
Insula						
Insula Anterior	R	3.35	26	16	−20	5.52
Sub-Cortical						
Amygdala	L	3.29	−30	2	−20	1.448
Putamen	R	3.72	28	−10	4	4.408
Thalamus (pulvinar)	R	3.35	18	−26	4	1.088
Brainstem/Cerebellum						
Cerebellum Crus2	L	3.28	−26	−78	−40	2.08
Cerebellum Crus2	R	3.72	4	−80	−36	1.408
Cerebellum Crus2	R	3.24	18	−82	−46	2.008
***Nalb-Sal>Nalb-Nalox***						
Cortical						
Frontal						
Superior Medial	L	4.17	−10	32	38	1.328
Superior Medial	L	3.12	−6	32	34	0.912
Superior Medial	L	3.95	−8	28	36	1.664
Middle	L	4.06	−30	4	50	9.712
Parietal						
Postcentral	R	2.98	58	−20	38	0.872
Occipital						
Rolandic Operculum	R	3.74	42	0	16	1.216
Rolandic Operculum	R	3.77	42	−4	16	0.424
Sub-Cortical						
Caudate	L	4.15	−18	−6	24	1.448
Caudate	L	3.77	−18	−16	24	0.944
Caudate	R	−4.45	14	18	16	4.344
Brainstem/Cerebellum						
CN V main sensory n.	R	4.02	2	−28	−38	1.056
CN V main sensory n.	L	4.07	−6	−28	−36	2.408
Pons	R	3.51	6	−26	−34	0.616
Cerebellum Crus2	R	3.17	38	−78	−42	3.592

Regions in which there were significant *differences* in activity induced by the two treatments without regard to the absolute value of the BOLD signal in these regions. Thus, a region may show a significant contrast even in the absence of significant activation or deactivation by either of the treatments. Regions where Nalb-Nalox induced significantly greater activity than Nalb-Sal are shown in the top section of the table; regions where Nalb-Sal induced significantly greater activity than Nalb-Nalox are shown in the lower section of the table. See caption for Table 1 for explanation of the columns. See Fig. 3 (“Contrast Maps: Nalb-Sal vs Nalb-Nalox”) for maps depicting the data in this table.

#### Effects of Sal-Nalb on brain activation

Increased BOLD responses were observed in 60 brain regions including, the occipital cortex, temporal cortex (mostly inferior), anterior and posterior insula, thalamus, caudate, hippocampus, pons, and cerebellum. Decreased responses were observed in 9 brain regions, including the middle frontal cortex, inferior orbitofrontal cortex, post central parietal cortex, superior temporal pole, and cerebellum (**Table 1** **, see** **Fig. 3** for maps). We interpret these changes to reflect *de novo* effects of nalbuphine.

#### Effects of Nalox-Nalb on brain activation

*Nalox-Nalb* produced a very different pattern of changes in activity; fewer regions showed increased activity (n = 14) and also fewer regions showed decreased activity (n = 3) compared to Sal-Nalb. Regions with increased activity included the lingual cortex, the anterior insula but not the posterior insula, and the cerebellum. Decreased responses were only observed in the inferior orbital region (**Table 2** **,** **Fig. 3**). Thus, based on infusion activation patterns, large differences were apparent between the Sal-Nalb and *Nalox-Nalb* sessions.

#### Differences in brain activation

Low-dose naloxone significantly blocked nalbuphine activation (i.e., Sal-Nalb>*Nalox-Nalb*) in superior medial and middle frontal cortex, postcentral parietal cortex (lateral aspect), occipital cortex (rolandic operculum), caudate, pons (trigeminal main sensory nucleus) and cerebellum (**Table 3** **,** **Fig. 3**). Addition of naloxone also induced activation of some areas significantly more than nalbuphine alone (i.e., *Nalox-Nalb*>Sal-Nalb); these included the superior and inferior orbital cortex (rostral), postcentral parietal cortex (superior aspect), occipital cortex (cuneus), temporal cortex (middle and superior poles), amygdala, putamen, pulvinar, and areas in the cerebellum.

### Functional connectivity

**Figure 4:**
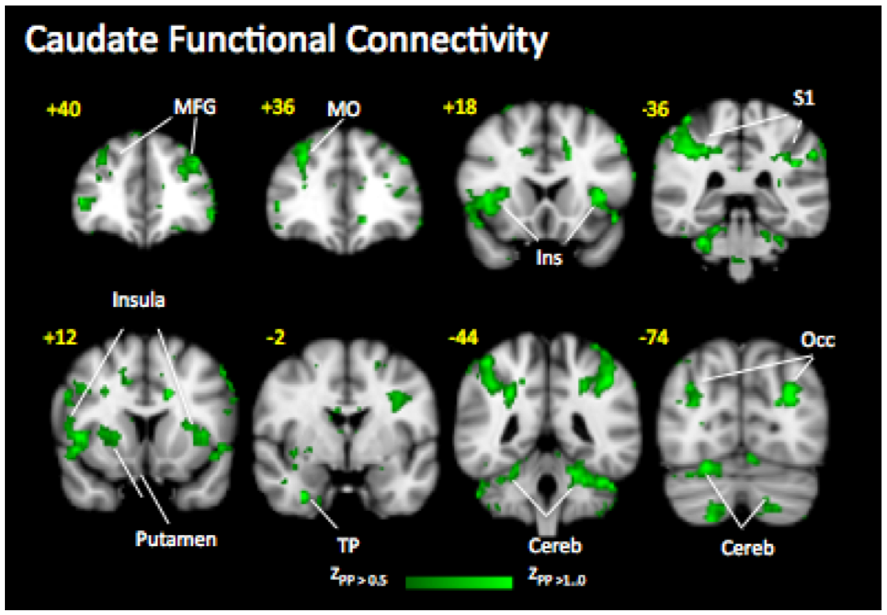
Functional Connectivity Maps. Caudate functional connectivity induced by Nalb-Sal that was blocked by naloxone (see Table 4). There was no functional significant connectivity induced by the Nalb-Nalox treatment.

Of the regions tested (e.g., thalamus, anterior insula, posterior insula, anterior cingulate, posterior cingulate, and caudate), only caudate was found to demonstrate significant Fc. Nalbuphine induced functional connectivity of the caudate with the following cortical areas: superior frontal, superior medial frontal, and middle frontal, middle orbitofrontal, inferior operculum, precentral frontal, superior and inferior parietal, calcarine and middle occipital, superior temporal and fusiform, middle cingulate, and anterior insula (**Table 4** **,** **Fig. 4**). Naloxone eliminated this connectivity between the caudate and all of these areas.

**Table 4:** Connectivity analysis for the caudate.

			Coordinates (mm)			Volume
Brain Region	Lat.	Zstat	X	Y	z	cm^3^
***Nalb-Sal>Nalb-Nalox***						
Cortical						
Frontal						
Superior Medial	L	2.82	−10	24	38	0.592
Superior	L	3.27	−18	10	54	0.496
Middle	L	2.82	−28	40	28	2.536
Middle Orbital	R	3.51	42	52	8	0.584
Middle Orbital	R	3.16	30	36	38	1.744
Middle Orbital	R	2.73	30	32	32	0.608
Middle Orbital	R	3.28	30	24	38	0.68
Inferior Orbital	R	2.65	50	24	−4	0.696
Inferior Orbital	R	2.70	46	18	−14	0.528
Inferior Orbital	L	3.36	−46	16	−10	0.976
Inferior Triangular	R	3.26	48	40	4	1.000
Inferior Operculum	R	3.05	50	8	16	0.608
Inferior Operculum	R	2.67	54	8	8	0.496
Inferior Operculum	L	2.85	−46	8	4	0.48
Inferior Operculum	R	2.96	48	6	26	0.624
Inferior Operculum	R	2.88	42	4	26	0.312
Precentral	R	3.37	44	6	32	1.376
Precentral	R	2.73	54	6	32	0.464
Precentral	L	3.68	−34	4	40	2.696
Parietal						
SupraMarginal	R	2.93	60	−24	32	1.336
SupraMarginal	R	4.33	54	−30	40	5.24
Postcentral	R	3.42	28	−36	40	0.472
Inferior	L	3.11	−56	−36	38	1.848
Inferior	R	2.87	44	−38	48	0.992
SupraMarginal	R	2.93	42	−42	40	0.784
Superior	R	3.13	46	−44	56	1.608
Inferior	L	3.35	−44	−44	44	1.696
Inferior	L	2.84	−46	−46	52	1.168
Inferior	R	2.79	42	−48	46	0.424
Angular	R	2.65	30	−52	44	2.728
Superior	R	3.26	20	−62	64	2.8
Superior	L	2.86	−30	−64	58	1.016
Occipital						
Middle	L	2.94	−26	−66	30	0.504
Middle	R	3.23	36	−70	30	0.704
Middle	L	4.07	−30	−74	26	2.144
Middle	R	2.65	32	−76	22	1.536
Middle	R	2.79	34	−78	34	0.56
Calcarine	R	3.03	22	−80	8	0.304
Middle	L	3.46	−32	−82	32	1.064
Temporal						
Superior	L	2.99	−44	2	−8	0.328
Fusiform	R	3.41	32	−2	−36	0.456
Fusiform	R	3.25	28	−32	−24	0.36
Cingulum						
Middle	R	2.77	12	24	36	0.416
Insula						
Anterior	R	2.97	40	20	−4	0.576
Anterior	L	3.54	−38	16	4	2.272
Anterior	R	3.57	44	14	2	2.144
Anterior	R	3.32	48	14	−6	0.888
Sub-Cortical						
Putamen	R	2.91	30	16	6	2.04
Cerebellum						
Cerebellum 4 5	L	2.8902	−22	−26	−28	0.624
Cerebellum 4 5	R	2.9845	20	−34	−22	0.392
Cerebellum 4 5	R	2.8301	20	−38	−22	0.24
Cerebellum 4 5	L	3.3849	−18	−42	−24	1.472
Cerebellum 4 5	R	3.0935	22	−46	−20	2.176
Cerebellum 4 5	L	2.9436	−18	−50	−26	1.032
Cerebellum 6	R	3.6119	32	−36	−32	1.376
Cerebellum 6	L	2.8626	−28	−42	−30	0.656
Cerebellum 6	L	2.7682	−32	−42	−30	0.376
Cerebellum 6	L	3.3063	−42	−44	−30	0.936
Cerebellum 6	L	2.8028	−26	−50	−28	0.784
Cerebellum 6	R	2.6501	24	−60	−26	0.96
Cerebellum 6	L	2.9134	−6	−62	−10	1.32
Cerebellum 6	R	2.7873	38	−64	−26	0.968
Cerebellum 6	L	2.8994	−12	−64	−18	2.32
Cerebellum 8	L	3.2813	−16	−66	−42	1.056
Cerebellum 6	R	2.8994	26	−66	−24	0.552
Cerebellum 6	R	3.073	16	−70	−20	2.36
Cerebellum 6	R	2.6594	34	−72	−24	0.248
Cerebellum 6	R	3.5358	24	−74	−18	1.048
Cerebellum Crus1	L	2.9171	−42	−50	−32	0.28
Cerebellum Crus1	R	2.7828	54	−54	−34	1.08
Cerebellum Crus1	L	3.4277	−52	−56	−32	1.376
Cerebellum Crus1	R	2.6522	52	−60	−28	1.168
Cerebellum Crus1	L	2.9529	−34	−68	−26	1
Cerebellum Crus2	L	2.9329	−38	−58	−44	1.12
Cerebellum 7b	L	2.6767	−16	−78	−44	0.472

Regions demonstrating significant functional connectivity to the caudate. Note that only Nalb-Sal showed significant connectivity. See caption for Table 1 for explanation of the columns. See Fig. 4 for maps depicting the data in this table.

## Discussion

In this study we investigated the effect of nalbuphine on brain circuitry implicated in analgesia and pain. For this analysis we used phMRI to identify changes in BOLD activity following infusion of nalbuphine with and without naloxone pretreatment. Nalbuphine preceeded by saline (i.e., Sal-Nalb) administration significantly increased activity in 60 discreet brain regions and decreased it in 9 (**Table 1**); nalbuphine pretreated with naloxone (i.e., Nalox-Nalb) significantly increased activity in only 14 regions and decreased it in only 3 (**Table 2**). Given that nalbuphine is an opioid agonist and naloxone an opioid antagonist, these results should perhaps not be surprising; naloxone blocked many actions of nalbuphine. These results might help explain nalbuphine's pain-facilitating effect when administered alone and its analgesic effect when pretreated with naloxone. Importantly, although pain measures are not presented in the present study, the relevance of the various identified brain regions to analgesia/algesia has been well established in previous studies (see **Table 5**).

**Table 5:** Comparison with drug actions in other studies.

Region	Subregion	Pain	Nalb-Sal		Nalb-Nalox			Contrasts	
			Activ.	Connect.	Activ.	Morph	Nalox	NN>NS	NS>NN
Frontal	Superior	+		1	−1			2	
	Superior medial	+		1					3
	Middle		−1	1					1
	Superior orbital						1		
	Middle orbital			4					
	Inferior orbital	+	−1	3	−1	−2	+	1	
	Inferior triangular			1					
	Inferior operculum			5					
	Precentral			3					
Occipital	Rolandic operculum		+3					2	
	Calcarine		+2	1					
	Middle		+2	6					
	Superior			−1					
	Cuneus						1		
Temporal	Superior pole	+	+1 −2			+	2		
	Middle pole	+				+	1		
	Superior	+	+2	1					
	Inferior	+	+4						
	Fusiform	+	+1	2					
	Lingual			+1					
	Superior lobe						1		
Parietal	Postcentral (S1, superior)	+	−2	1				1	
	Postcentral (S1, lateral)	+						1	
	Precuneous	+	−1						
	Supramarginal			3					
	Superior			3					
	Inferior			5					
	Angular			1					
Insula	Anterior	+	+4	4	+5			1	
	Posterior	+	+3			+			
Cingulate	Middle	+		1			+	1	
Sub-cortical	Thalamus	+	+1		−2				
	Pulvinar		+2				1		
	Caudate	+	+1					3	
	Putamen			1	+1	+2		1	
	Nucleus accumbens	-			+2				
	Amygdala						1		
	Substantia nigra				+1				
	Hypothalmus				+1				
	Sublenticular extension				+1				
	Hippocampus	+	+7		+2	+			
	Pallidum	+							
	PAG	+				+			
	Pons	+	+4					1	
	spV			1					
	Main sensory n. CN V		+1					2	
Cerebellum	+	+22 −2	27	+6			3	1	
**Totals**			**+60 −9**	**74**	**+14 −3**	**+9 −4**			

This table summarizes the data from the present study and three earlier studies in healthy male volunteers. Plus signs (+) indicate brain regions showing significant activation; minus signs (−) indicate brain regions showing significant deactivation. Numbers indicate count of discreet brain sites within each named subregion.

Columns:

“*Pain*” Brain regions showing statistically significant BOLD signal changes in our study of noxious stimulation [42]. Note that the count of discrete brain sites within each named subregion is not shown here.

“*Nalb-Sal.*” Consists of two subcolumns “*Activ.*” and “*Connect.*”

“*Activ.*” Brain regions showing statistically significant BOLD signal changes in response to saline followed by nalbuphine (Table 1).

“*Connect.*” Brain regions showing statistically significant functional connectivity with respect to caudate (Table 4). Numbers indicate count of discreet brain sites within each named subregion showing functional connectivity. Note that this only occurred in the Nalb-Sal group; no significant connectivity was observed in the Nalb-Nalox group.

“*Nalb-Nalox*” Brain regions showing statistically significant BOLD signal changes in response to naloxone followed by nalbuphine (Table 2).

“*Morph*” Brain regions showing statistically significant BOLD signal changes in our study of morphine [8].

“*Nalox*” Brain regions showing statistically significant BOLD signal changes in our study of naloxone (Borras et al., 2004). Note that the count of discrete brain sites within each named subregion is not shown here.

“*Contrasts*” Consists of two subcolumns “*NN>NS*” and “*NS>NN.*” The numbers indicate the count of discreet sites demonstrating significant *differences* in BOLD activation/deactivation between the two treatments (Table 3); therefore, individual sites in may or may not be found in Tables 1 or 2.

“*NN>NS*” Regions where naloxone followed by nalbuphine showed significantly greater activation than saline followed by nalbuphine.

“*NS>NN*” Regions where saline followed by nalbuphine showed significantly greater activation than naloxone followed by nalbuphine.

All studies represented in the table were performed using the 3T Siemens scanner, and the studies for morphine and naloxone used the same infusion protocol as the current study.

### Actions of Nalbuphine (Nalb-Sal)

Because nalbuphine is widely employed as an analgesic, but also produces a marked pain-facilitation in males, its phMRI brain signature might be expected to show characteristics typical of both analgesia and pain, and this is indeed the case. Nalbuphine acted at sites associated with the production of analgesia in that it attenuated activity in some brain regions in which activity was also attenuated by morphine (see **Table 5**; data from our prior study [8]). However, it also activated many areas that are activated by noxious stimulation, areas that were not activated by either morphine or nalbuphine pretreatment with naloxone. Like morphine, nalbuphine attenuated activity in the inferior orbital cortex, but, like noxious stimulation, it increased activity in several regions in the temporal cortex, insula, thalamus (including pulvinar), caudate, and pons. In contrast to noxious stimulation, nalbuphine decreased activity in the primary somatosensory cortex (S1) and precuneous regions. In addition, nalbuphine induced functional connectivity of the caudate and multiple regions in the frontal, occipital, temporal, insular, middle cingulate cortices, the putamen, and many areas in the cerebellum.

### Actions of Nalbuphine in the Presence of Naloxone (Nalb-Nalox)

Fewer brain regions were activated when nalbuphine was administered with naloxone, down from 60 to 14, and also fewer regions were deactivated, down from 9 to 3 (**Table 5**). Given that naloxone abolishes nalbuphine-induced pain-facilitation and induces significantly enhanced analgesia [4], these results suggest that naloxone blockade affects activity in areas that mediate pain facilitation. Consistent with this suggestion, regions in which naloxone blocked activity included pulvinar, which has been associated with increased activation in central sensitization [21], pons, which has been implicated in remifentanil withdrawal hyperalgesia [22], posterior insula, which is associated with pain-exclusive activations [23], and many areas in the cerebellum, which may have a role in pain processing [24]. Connectivity analysis showed that naloxone reduced nalbuphine-induced functional connectivity of caudate with all regions; that is, there was no significant connectivity in the presence of naloxone. Interestingly, some structures with reduced caudate connectivity (putamen, cingulate, temporal lobe, and cerebellum) showed significantly greater BOLD activation (Tables 3, 5). Increased activation of putamen was also observed with morphine [8], consistent with a role of this region in analgesia [25]. These results suggest that nalbuphine-induced activation of caudate (perhaps by disinhibition, as opioids are inhibitory) initiates pain-enhancing connectivity with other regions, and, although the receptor(s) involved is not known, blockade of this connectivity by naloxone abolishes this pronociceptive effect.

### Drug Modulation of Interoception

The insula is postulated to be involved in interoception or a state of subjective feeling, defined as *sensitivity to stimuli originating inside of the body* [26]. The region, specifically the mid/posterior insula is connected with the posterior middle cingulate cortex (pMCC) that may “integrate interoceptive information with emotional salience to form a subjective representation of the body” [27]. Such ongoing subclinical processing may be altered by naloxone-nalbuphine vs. saline-nalbuphine effects. In essence, changes akin to those surmised in drug addiction may be present, albeit acutely [28]. A potential mechanism of the changes observed here (i.e., naloxone-induced inhibition of the insula cortex) may relate to naloxone effects on opioid receptors in the insula. The insula has high concentrations of opioid receptors [29] and naloxone may act to block an opioid-induced effect – i.e., the nalbuphine effects on the posterior insula. In support of this, naloxone infusion in healthy subjects, as measured by arterial spin labeling showed increases in insula blood flow following fentanyl, but not naloxone [30]. In a similar manner, the effect of naloxone may be to decrease effective action of nalbuphine.

### Time course of effects

In males nalbuphine by itself induces early onset analgesia that descends into anti-analgesia between 40 and 90 minutes after administration, depending on dose [3]. For the 5 mg dose of nalbuphine, the approximate dose used in the current study, the duration of analgesia is shorter—about 30 minutes—and the appearance of anti-analgesia is earlier than for higher doses [3]. Thus, since the plasma half-life of naloxone is about an hour [31], the timing of the effects of naloxone observed in the present study are consistent with our clinical observations. Of note, it is likely that nalbuphine alone activates both analgesia and algesia mechanisms at the same time and that addition of naloxone eliminates that effect on the algesic circuitry.

### Brain Generated Pain Enhancement

An important implication of these findings is that the brain can generate *de novo* pain enhancement elicited by the action of a drug such as nalbuphine. Aside from the clinical data arising from lesions of the nervous system (e.g., thalamic or brainstem stroke), few studies have investigated pain facilitation in the brain. Derbyshire and colleagues [32] showed that hypnotically-induced pain is accompanied by activation of classic pain regions, including anterior cingulate cortex (ACC), insula, and prefrontal cortex. Kong and colleagues [33] found that an expectation/conditioning manipulation model can produce a nocebo effect (expectation-induced pain enhancement, opposite of the placebo effect) correlated with activation of the medial pain system. Remifentanil offset or withdrawal is associated with acute opioid withdrawal hyperalgesia with increased activation in the mesencephalic pontine reticular formation [22]. We previously reported that naloxone alone, in contrast to its effect on nalbuphine-induced enhancement in pain circuits in the present study, increased brain activation induced by noxious thermal stimulation [9]. Thus, the brain appears to contain circuitry necessary for pain *facilitation*, a phenomenon distinct from pain induction or transmission, as nalbuphine alone does not induce pain.

Direct interventions in pain pathways can modulate nociceptive responses mediated by descending pathways. These include both non-opioid and opioid-based manipulations. Calejesan and colleagues [34] showed that either electrical stimulation or microinjection of a metabotropic glutamate receptor agonist into ACC facilitated the nociceptive tail-flick reflex (i.e., enhanced nociception), and these effects were mediated through the rostral ventral medulla (RVM). In contrast, electrical stimulation of the ACC reduces responses of dorsal horn neurons to noxious mechanical stimuli, suggesting an analgesic effect [35]. Although the findings of these studies disagree in terms of polarity, they agree that manipulation in the ACC, an important region in pain processing, can influence nociceptive responses at the level of the spinal cord. Moreover, there is evidence of connectivity between the ACC and the descending pain modulation system. Neural afferent projections from ACC to the periaqueductal gray (PAG) have been reported [36], [37], and functional connectivity based on fMRI data from 100 subjects has been reported between ACC, PAG, and RVM [38]. Interestingly, this study, which included equal numbers of males and females, also found sexually dimorphic functional connectivity at the mid-cingulate cortex. Others have reported sex differences in brain processing in a number of regions, including the perigenual cingulate cortex, in a study of equal pain experience induced by laser stimulation [39]. Whether these differences contribute to the sex differences in analgesic efficacy observed in our clinical studies [1], [2], [3], [4] remains to be investigated.

### Limitations

A few caveats of this study need to be considered. (i) Although 12 subjects per cohort is a reasonable number for phMRI studies [40], smaller effects are probably not statistically robust enough to be detected. (ii) We did not observe significant differences in functional connectivity, the results probably were affected by the use of whole anatomical areas, rather than specific areas associated with significant differences. In order to use ROIs defined by statistical differences a different cohort of subjects would have been needed at it is not statistically correct to use the same cohorts to derive an ROI for secondary functional connectivity analysis. (iii) Prolonged effects on brain systems by low single dose naloxone is futher supported by prolonged (for hours) opioid receptor binding (as measured by [^11^C]-carfentanil displacement by naltrexone) [41]. In addition in our prior study of naloxone infusions in healthy men (albeit at higher doses), effects on brain system activation was observed within 10 minutes following the infusion and later (i.e., >20 min following the infusion) subjective measures of pain intensity and unpleasantness revealed significant differences even though no significant effects on BOLD were observed approximately 10 minutes after the initial infusion [9]. (iv) We did not perform a dose-response/multiple dose study across healthy subjects and patients that may provide insights into differences in response between the two groups. Such phMRI studies would help differentiate the effects of clinical pain on the phMRI signal. (v) Future studies evaluating effects in healthy and clinical groups of men and women would be needed to further dissect the pharmacological and psychometric differences across gender.

### Conclusions

This study provides support for the use of phMRI in our understanding of the mechanism of action of CNS-acting analgesics and the development of more selectively targeted agents. The brain activity signature of nalbuphine demonstrates characteristics typical of both noxious stimulation and the opioid analgesic morphine, thus providing a neural basis for its paradoxical ability to produce both analgesia and pain-facilitation. Naloxone selectively blocks the actions of nalbuphine in brain regions associated with pain, leaving the analgesic-like actions intact. Given these findings, and that nalbuphine alone does not produce pain, it is possible that nalbuphine interacts with a pain salience system, which can modulate perceived pain intensity relative to the nature of the initial insult.

Although nalbuphine is the κ-agonist antagonist we have studied most extensively [2], [3], [4], we have shown that the other two clinical agents in this class, pentazocine and butorphanol, behave similarly [1], [2], strongly implying similar mechanisms of action of this class of opioid drugs. All three drugs in this class are known to act at multiple receptors; therefore, it will be important to identify the specific receptors mediating pain enhancement compared to those that mediate analgesia, in order to develop more efficacious analgesic treatments.
